# A hybrid simulator model for the control of catastrophic external junctional haemorrhage in the military environment

**DOI:** 10.1186/s41077-016-0008-z

**Published:** 2016-02-09

**Authors:** Katarina Silverplats, Anders Jonsson, Lars Lundberg

**Affiliations:** 1 Swedish Armed Forces Centre for Defence Medicine, Gothenburg, Sweden; 2 Department of Orthopaedic Surgery, Sahlgrenska University Hospital, Gothenburg, Sweden; 3 Centre for Prehospital Research, A2, University of Borås, Borås, Sweden

**Keywords:** Extremity injuries, Haemorrhage control, Haemostatic agents, Junctional haemorrhage, Military, Prehospital training

## Abstract

Catastrophic haemorrhage from extremity injuries has for a long time been the single most common cause of preventable death in the military environment. The effective use of extremity tourniquets has increased the survival of combat casualties, and exsanguination from isolated limb injuries is no longer the most common cause of death. Today, the most common cause of potentially preventable death is haemorrhage from the junctional zones, i.e. the most proximal part of the extremities, not amenable to standard tourniquets.

Different training techniques to control catastrophic haemorrhage have been used by the Swedish Armed Forces in the pre-deployment training of physicians, nurses and medics for many years. The training techniques include different types of human patient simulators such as moulage patients and manikins. Preferred training conditions for the control of catastrophic haemorrhage include a high degree of realism, in combination with multiple training attempts.

This report presents a new hybrid training model for catastrophic external junctional haemorrhage control. It offers a readily reproducible, simple and inexpensive opportunity to train personnel to deal with life threatening catastrophic junctional haemorrhage. In particular, this model offers an opportunity for non-medical military personnel in Sweden to practice control of realistic catastrophic haemorrhage, with multiple training attempts.

## Introduction

### The medical challenge

Most battlefield casualties die of their injuries in the pre-hospital environment. Approximately 25 % of these are potential survivors, if early and effective treatment is provided [1–3]. Uncontrolled haemorrhage is the most common preventable cause of death for wounded soldiers in military combat trauma [4–9]. Hypovolemic shock caused by significant blood loss can lead to acute coagulopathy, hypothermia and acidosis, called “the lethal triad” [10]. Ultimately, there is a higher risk of morbidity and late mortality due to sepsis and multiple organ failure [11]. Exsanguination from extremity wounds accounts for over 30 % of all preventable deaths, and injuries to the junctional zones are responsible for about 20 % of all uncontrolled haemorrhage [1, 12]. The term “junctional” is an established term to describe haemorrhage locations just outside the thorax and abdomen, where tourniquets are not possible to apply. Examples of junctional zones are the most proximal parts of the extremities, namely the groins and axillae.

With the widespread and successful use of the extremity tourniquet, isolated limb exsanguination is no longer the most common cause of preventable death on the battlefield – the commonest cause is now groin haemorrhage amenable to truncal tourniquets [13, 14]. Junctional zone wounds are unsuitable for extremity tourniquet application and present a particular challenge for medical prehospital personnel [7, 14, 15].

### Pre-hospital treatment of catastrophic haemorrhage

The use of extremity tourniquets to control catastrophic haemorrhage has been known for many centuries, but the popularity of the method has varied greatly [13, 16–18]. It is known that a tourniquet applied with high pressure for more than a couple of hours may cause severe tissue damage [19, 20]. With the increased use of Improvised Explosive Devices (IED) in recent warfare, the number and severity of traumatic limb amputations with subsequent catastrophic haemorrhage has multiplied. This new injury pattern, and the development of effective tourniquets, is a major reason behind the renewed popularity of the tourniquet. The widespread use of effective extremity tourniquets, in combination with novel haemostatic agents, has decreased the incidence of death from exsanguination due to extremity injuries and non-compressible wounds [5–8, 21, 22], though the reasons for improved survival are multi-factorial [23].The new challenge is to control haemorrhage from body areas too proximal for extremity tourniquets. Non-treated junctional injury is common, severe, disabling and lethal [24]. Different kinds of junctional emergency tourniquets have been developed and evaluated for prehospital use [14, 25–27].

### Need for education and training

It is important for military medical personnel, and indeed all soldiers, to understand their options for the control of haemorrhage and how to master these techniques. One of the challenges is to prepare all first responders, who usually are non-medical personnel, for the time-critical injuries they will encounter on the battlefield. Preferred training conditions for the control of catastrophic haemorrhage include a high degree of realism, in combination with multiple training attempts. In this report, different models for training of haemorrhage control in extremities and junctional zones are presented and discussed, including a new and inexpensive hybrid training model specifically constructed for the control of catastrophic external junctional haemorrhage. The report was initiated and approved by the Swedish Armed Forces Animal Welfare Body.

## Training models for the control of extremity and junctional zone haemorrhage

From an international perspective, different training models have so far included traditional human patient simulators [28], a haemorrhage simulator [9], animal models for Live Tissue Training (LTT), [4, 29–31], and an alternative fresh meat model to simulate life-threatening external bleeding without recourse to live animal training [32].

In Sweden, military medical personnel are trained how to respond to the most common cause of preventable battlefield fatalities, including extremity and junctional zone haemorrhage. We use different kind of techniques to practice these real-life trauma scenarios, such as traditional moulage scenarios involving simulated patients, computerized patient simulators, Live Tissue Training (LTT), and a new hybrid model designed to practice the treatment of catastrophic junctional haemorrhage.

### Traditional moulage scenarios involving simulated patients

The use of moulage patients, with actors dressed as casualties with simulated injuries and acting correspondingly, has for decades been a standard procedure in military medical training. This method is to a high degree dependent on the acting abilities of the moulage patient.

### Computerized patient simulators

The Swedish Armed Force Centre for Defence Medicine (SWE CDM) has used computerized human patient simulators for medical training since about 2000. When the SWE CDM relocated to Gothenburg in 2006, a simulation lab was built. The University of Gothenburg has also used this simulation lab for the training of medical students. At present, we use third generation wireless manikins (SimMan 3G®) in the simulation lab.

### Live Tissue Training (LTT)

The Swedish Armed Forces to some degree use anesthetized pigs in combat medical training for special target groups such as physicians, nurses, medics and other personnel with delegation from HQ to perform specified operations. Special licenses and ethical approval are needed to use live animals for this purpose, and premises for keeping animals have to be approved regarding size, climate, equipment and security. Prior to every LTT session, participants attend a mandatory ethical lecture. The need for LTT is evaluated for every exercise. During the animal exercise there is always a veterinarian and anaesthesia/Intensive Care nurse present making sure that the animals are properly anaesthetized and haemodynamically stable.

By using LTT, military medical personnel may practice the chain of medical care from point of wounding to the field hospital level, including emergency management of internal and external catastrophic haemorrhage.

### A hybrid model for catastrophic external junctional haemorrhage

**Fig. 1:**
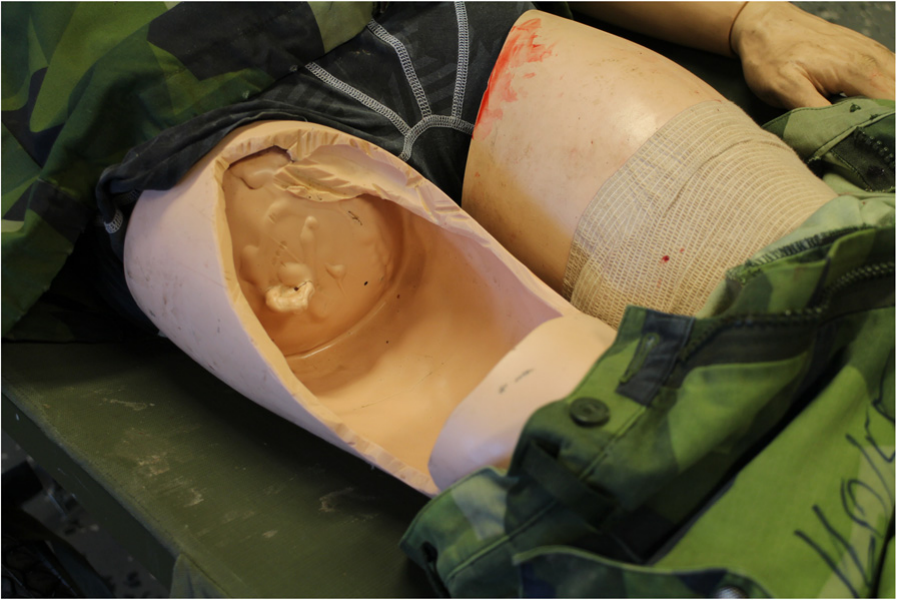
In the area of the groin and proximal thigh, the plastic surface is removed to create a cavity where the haemorrhage model is placed

**Fig. 2:**
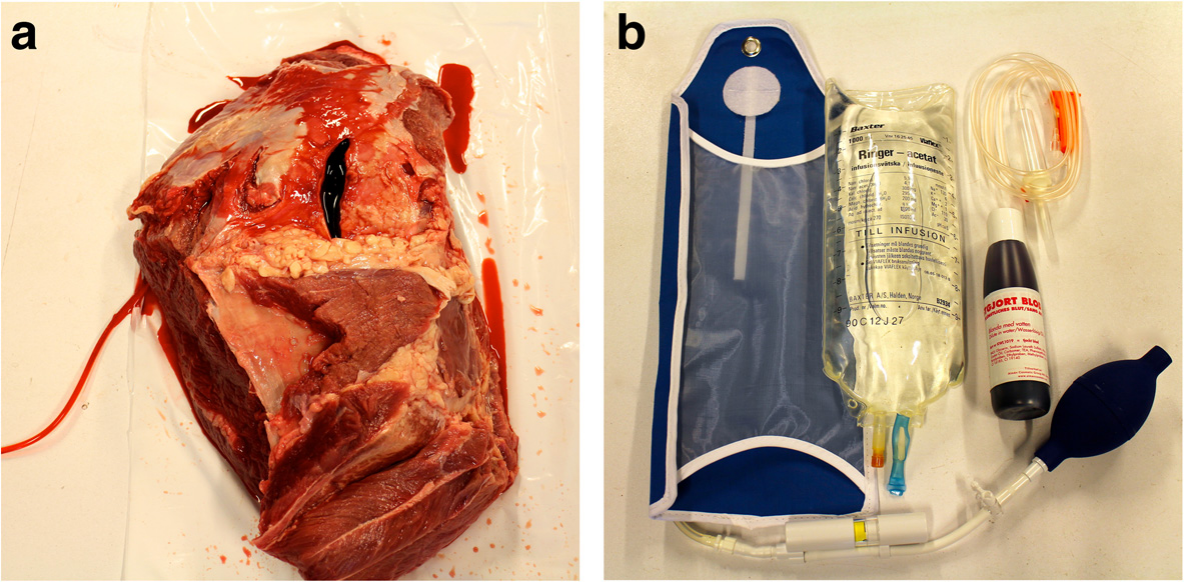
The haemorrhage model is made of a slab of meat, consisting of an injury made by a scalpel, with a "bleeding vessel" made of a Foley catheter connected to a bag of red coloured warm Ringer Lactate to simulate blood (2a). To simulate arterial catastrophic haemorrhage, the bag with artificial blood is placed in a pressure bag, which is managed by the instructor (2b)

**Fig. 3:**
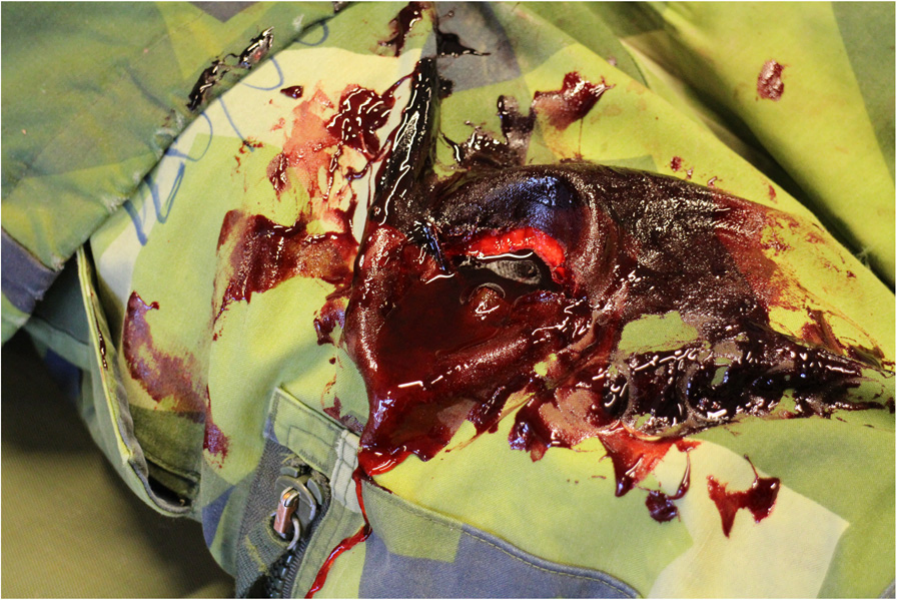
For realism, the manikin is dressed as a soldier and can be placed in an environment similar to the military battlefield

At SWE CDM we have developed a hybrid model based on a realistic model made for external haemorrhage training by the British Defence Medical Services, originally described by Moorhouse et al. [32]. The Swedish hybrid model consists of a human sized plastic training manikin, which has been taken out of use from the simulation lab after many years of service. In the area of the groin and uppermost part of the thigh the plastic surface is removed to create a cavity where the haemorrhage model is placed (Fig. 1). The haemorrhage model consists of a slab of meat, with an injury made by a scalpel, containing a "bleeding vessel" made of a Foley catheter connected to a bag of red coloured warm Ringer’s Lactate to simulate blood (Fig. 2a-b). To simulate arterial catastrophic haemorrhage, the bag with artificial blood is placed in a pressure bag managed by the instructor. During drill, the student first has to apply effective manual pressure above the injury site (proximal control) and after that pack the wound cavity with haemostatic gauze (QuikClot Combat Gauze®, Celox Gauze®) to stop the bleeding. For realism, the manikin is dressed as a soldier and can be placed in an environment similar to the military battlefield (Fig. 3).

Before the student starts using the model, an instructor demonstrates how to apply manual pressure and haemostatic gauze to stop the bleeding and describes the haemostatic agents used, how they work and why it is important to stop or control the haemorrhage at the prehospital location.

## Discussion

Military medical personnel are expected to perform lifesaving procedures without having the same equipment as in civilian work, therefore it is important to give them the best possible training with a high degree of realism and also an opportunity to do multiple training attempts [30]. Without training programs including different methods for treating catastrophic haemorrhage most medics would be sent into a combat situation having never seen or treated a real traumatic injury [32].

### Traditional moulage scenarios involving simulated patients

The use of moulage patients will always be needed as a basic training method. It can be done either as simple role-playing or be supplemented with audio-visual simulation and professional acting. With the introduction of professional casualties, such as Amputees in Action®, where acting individuals have personal battlefield experience of being wounded, moulage has become a very powerful method. The main limitations here are the small number of professional actors available, and the inability to perform invasive or painful procedures on them.

### Computerized patient simulators

The benefits of simulation training are well established. It includes deliberate, repetitive practice in a safe and controlled environment. The simulation training is designed to simulate combat environments and real-life trauma scenarios [33–36]. Today, heavy-duty military manikins exist on the commercial market, and these manikins have been evaluated for an eventual purchase. They have a high degree of visual realism, but do not provide any realistic live tissue feeling when applying haemostatic gauze into the wound. They are also quite expensive.

### Live Tissue Training (LTT)

The challenges in using LTT for the training of non-medical personnel are the high number of trainees and the legal regulations used according to the principles of “3R" (Replace, Refine and Reduce), first described by Russell & Burch [37]. This means that LTT may only be used when there are no alternatives. The primary aim in a LTT exercise is to train doctors and nurses in surgical methods for control of external and internal catastrophic haemorrhage.

Many authors say that the major benefit of LTT is the realism it provides, since the animals are living beings [4, 38]. However, the few studies comparing LTT and simulator training show the benefits of simulation to be equivalent or superior to LTT [39, 40].

There is a need to replace LTT with other educational methods such as simulation. The growing public concern for animal welfare is not the only reason. The major disadvantage of LTT is the necessity for purpose built facilities with a high degree of security and legal restrictions [41]. In many countries LTT is not allowed. Only six of the 28 NATO member nations still use live animals for the training of combat medics [42].

In the Swedish Armed Forces, LTT is used in close adherence to the previously described principles of “3R”. We replace LTT with other training methods whenever possible; optimize the use of every single animal when LTT is considered necessary; and continuously try to reduce the total number of animals used. It is our belief that LTT should not be used as a basic training, but perhaps for final evaluation.

### A hybrid model for catastrophic external junctional haemorrhage

The simulator model developed represents the most common location for a catastrophic external junctional haemorrhage in the military environment, i.e. the most proximal part of the lower extremity. It is also easy to modify the manikin in this particular region. It would be desirable to modify the manikin in other junctional areas, such as perineum, axillae and neck. However, from a technical point of view these modifications are more difficult to do.

In the basic training for control of catastrophic haemorrhage, realistic haemorrhage models provide a better alternative than LTT. For this basic training, we choose to use warm red coloured Ringer’s Lactate to simulate blood rather than expired human blood or fresh animal blood. Our initial experience suggests that our hybrid model is a good alternative for both medical and non-medical personnel as a first step to practice control of external junctional catastrophic haemorrhage. The model has been used since 2012 in our medical training programme, in particular for combat life savers. It offers a reproducible, simple and inexpensive opportunity to train personnel to deal with life threatening catastrophic junctional haemorrhage. In particular, it offers an opportunity for non-medical military personnel to practice control of such haemorrhage, and to practice repeatedly until they are both competent and confident in these skills.

Further studies are needed in order to evaluate the validity of this hybrid model in basic training for the control of catastrophic haemorrhage. Such studies should include independent third party assessment of students’ ability to control catastrophic haemorrhage and to what extent has the simulator training affected their decision-making and manual skills.

## Conclusions

Out of the number of different training techniques for the control of catastrophic bleeding in the military environment, no single method can yet be described as the golden standard. The hybrid model presented in this paper is suggested as a readily reproducible, simple and inexpensive opportunity for both medical and non-medical military personnel to become and remain competent in controlling the most common form of catastrophic junctional haemorrhage.
